# Bilateral Breast Irradiation Using Hybrid Volumetric Modulated Arc Therapy (h-VMAT) Technique: A Planning Case Report

**DOI:** 10.7759/cureus.914

**Published:** 2016-12-05

**Authors:** Sitaraman Balaji Subramanian, Karunakaran Balaji, Moorthi Thirunavukarasu, Sumana Premkumar

**Affiliations:** 1 Department of Radiation Oncology, Global Hospitals, Chennai

**Keywords:** bilateral breast cancer, vmat, hybrid vmat

## Abstract

The purpose of this planning case report is to share the perceived dosimetric benefits of innovative hybrid volumetric modulated arc therapy (h-VMAT) for bilateral breast cancer radiotherapy in two patients with synchronous bilateral breast cancer. Two patients with early bilateral breast cancer after breast conservation surgery and adjuvant chemotherapy were planned for bilateral breast radiotherapy. On the planning computed tomography (CT) dataset, bilateral breast planning treatment volume (PTV) and organs at risk (OARs) were delineated using standard guidelines. Using the same structure set, volumetric modulated arc therapy (VMAT) and h-VMAT plans were generated and compared dosimetrically. The h-VMAT showed comparable target coverage, conformity and homogeneity while sparing of both lungs and heart were better. The dose to heart was reduced with h-VMAT, with a V_25Gy _and V_5Gy _of 3.2 & 22.3% for h-VMAT versus 11.6 & 84.9% for the VMAT plan. Similarly, the dose to the total lung was better in h-VMAT with a V_20Gy_ and V_5Gy_ of 12.1 & 46.2 versus 19.9 & 83.3% for VMAT. Overall the results indicate a better sparing of lung and heart at low doses with h-VMAT. Long-term clinical follow-up will give us more insight about the dosimetric benefits of these innovative techniques.

## Introduction

Synchronous bilateral breast cancer is uncommon with an estimated incidence of 2.1% [1]. In patients diagnosed with early synchronous bilateral breast cancer, breast conservation therapy is feasible [2]. Planning for bilateral breast cancer radiotherapy has always been a challenging task. Conventional radiotherapy uses tangent pair setup with a field-in-field technique that has significant issues like field overlap and suboptimal planning target volume (PTV) coverage. The purpose of this planning case report is to share the perceived dosimetric benefits of innovative hybrid volumetric modulated arc therapy (h-VMAT) over volumetric modulated arc therapy (VMAT) for bilateral breast cancer radiotherapy.

## Case presentation

Two consecutive patients diagnosed with synchronous bilateral early breast cancer at our institution were selected for this planning study. Both the patients underwent bilateral breast conservation surgery. Table 1 shows the basic characteristics of the two patients. After completion of adjuvant chemotherapy, they were referred for bilateral breast irradiation. This study was not a treatment-based study but a dosimetric planning case report. Patient information was anonymized to protect confidentiality.

**Table 1:** Basic characteristics of the two patients TNM = tumour, node and metastasis ER = estrogen receptor PR = progesterone receptor HER-2/neu = human epidermal growth factor (EGF) receptor-2

	Patient-1	Patient-2
Age (Years)	51	65
Pathological - Stage (TNM)		
Right breast	pT2pN0 (IIA)	pT2pN0 (IIA)
Left breast	pT1pN0 (IA)	pT2pN0 (IIA)
ER/PR status	Negative	Positive
HER-2/neu Status	Negative	Positive

The computerized tomography (CT) dataset of these two patients, who were immobilized using vacuum bag in a supine position with both the arms above the head, was used for planning purpose. CT scans were done with a slice thickness of 2.5 mm during normal breathing. In the first step, the PTV (right and left breast) was delineated in accordance with guidelines proposed by the Danish Breast Cancer Cooperative Group [3]. Organs at risk (OARs) contoured were both lungs and heart. In the second step, VMAT and h-VMAT plans were generated with a prescription dose of 50 Gy in 25 fractions. Eclipse (V-10, Varian Medical Systems-USA) treatment planning system (TPS) was used for all planning purposes. VMAT plan was done using three continuous arcs (arc length: 150^o^–210^o^) with 6 MV photon beams on Truebeam STx linear accelerator (Varian Medical Systems, USA). A single isocentre placed medially under the sternum was used for optimization. The collimator angle was set to a value of +10^o^. Interactive optimization objectives were used for each plan, keeping the dose to OARs as low as possible without compromising the PTV coverage. The h-VMAT planning involves two steps: first, field-in-field (FIF) forward planning setup with 80% of the prescription dose was planned for both the breasts. The heart and lungs were spared using the high definition multileaf collimator (HDMLC). Second, the remaining 20% prescription dose for both the breasts was optimized using three continuous arcs (arc length: 150^o^–210^o^) VMAT by keeping the dose delivered in FIF arrangement as the base dose plan. The final dose calculations were performed using anisotropic analytical algorithm (AAA) with 2.5 mm calculation grid size. All plans were normalized to 100% in target mean. Both planning techniques were evaluated using dose-volume histogram (DVH). PTV dosimetric parameters evaluated were PTV coverage (D_95%_), hot spot (D_2%_), conformality index (COIN) defined as (PTVref/PTV) x (PTVref/Vref), homogeneity index (HI) defined as (D_2%_ - D_98%_)/D_50%_ and total monitor units (MU).

In addition, h-VMAT plans were verified using portal dosimetric measurements (Varian Medical Systems, USA). TPS predicted and measured dose was analysed with three percent dose difference (DD) and 3 mm distance to agreement (DTA) criteria.

## Discussion

Table 2 shows the dosimetric results achieved in both the techniques for these two patients' CT-dataset.

**Table 2:** Dosimetric results achieved in both the techniques (VMAT &h-VMAT) VMAT = volumetric modulated arc therapy h-VMAT = hybrid-volumetric modulated arc therapy Gy = Gray COIN = conformality index HI = homogeneity index MU = monitor units Dmean = mean dose DX% = dose to X% of volume VXGy = volume receiving X Gy of dose

Dosimetric parameter	Patient-1		Patient-2	
	VMAT	h-VMAT	VMAT	h-VMAT
PTV				
D_95%_ (Gy)	47.5	47.6	47.6	48.5
D_2%_ (Gy)	52.2	51.4	52.4	51.4
COIN	0.84	0.82	0.81	0.80
HI	0.12	0.10	0.11	0.07
Heart				
Dmean (Gy)	10.5	5.0	16.2	5.4
V_25Gy _(%)	8.5	4.2	14.6	2.2
V_5Gy_ (%)	79.4	13.3	90.4	31.3
Total Lung				
Dmean (Gy)	12.1	8.3	13.8	10
V_20Gy _(%)	16.6	11.0	23.3	13.1
V_5Gy_ (%)	84	42.4	82.5	50
Total MU	761	612	716	557

**Figure 1:**
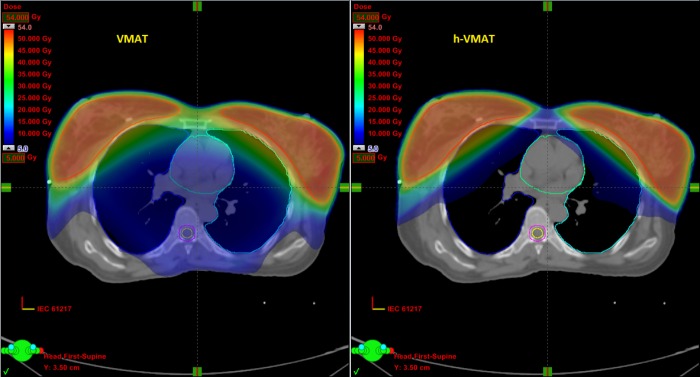
Comparative dose distribution in VMAT & h-VMAT VMAT = volumetric modulated arc therapy h-VMAT = hybrid-volumetric modulated arc therapy

PTV parameters like coverage, COIN and HI were similar in both the techniques. In both the techniques, hotspot in the overlap region was avoided. The h-VMAT achieved better sparing of heart at Dmean, V25Gy and V5Gy and lungs at Dmean, V20Gy and V5Gy. Total MU was comparatively less in h-VMAT. Figure 1 shows the low dose spread (V5Gy) in VMAT and h-VMAT plans.

**Figure 2:**
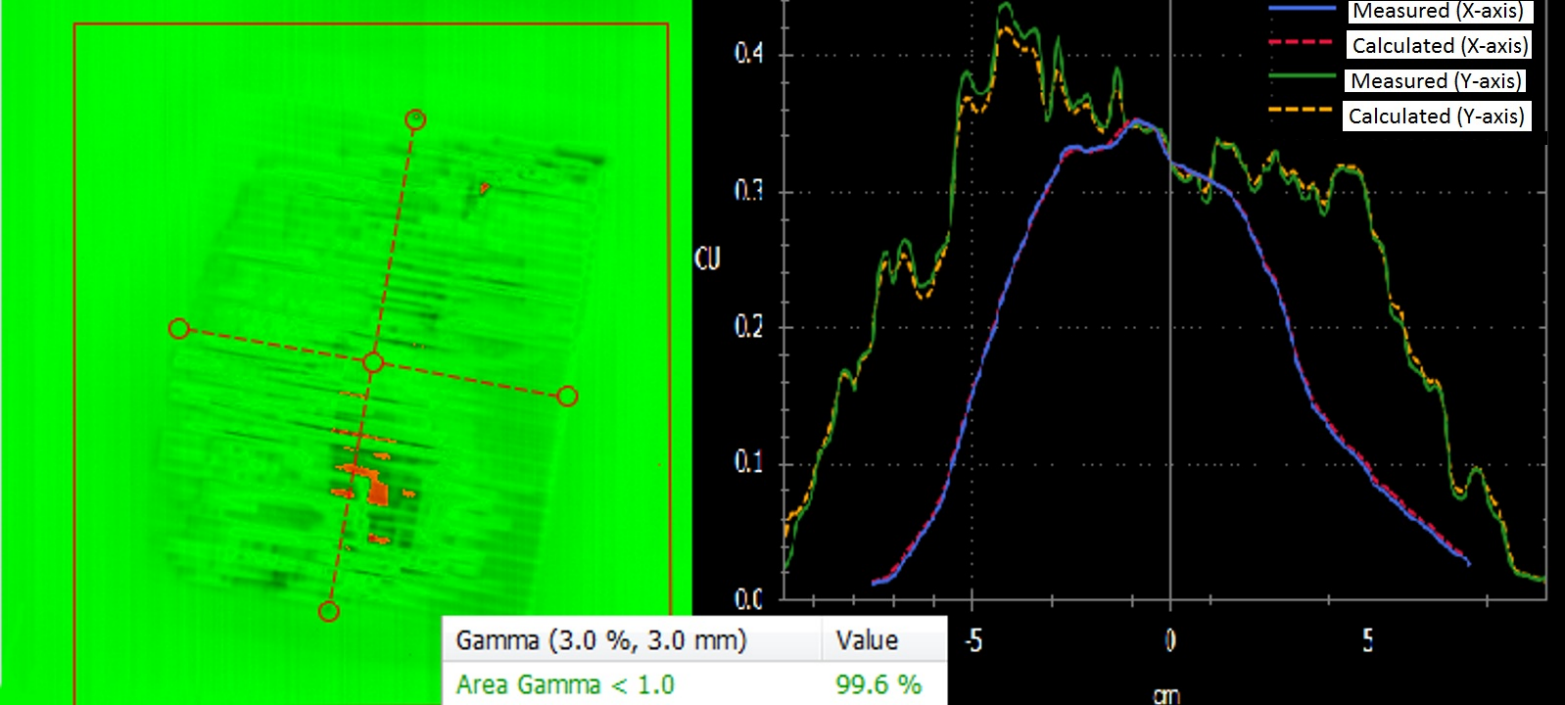
Gamma analysis for h-VMAT (portal dosimetry)

 Portal dosimetric measurements done for h-VMAT plans showed a gamma agreement greater than 97% (Figure 2).

In the literature, dosimetric studies using VMAT and tomotherapy have demonstrated the feasibility of delivering radiotherapy in bilateral breast cancer patients [4-5]. High-end conformal radiotherapy techniques improve conformality and avoid overdose at the overlap region at the cost of increased low dose spillage to the OARs. It was found that h-VMAT and hybrid intensity modulated radiation therapy (h-IMRT) used for breast cancer patients reduce low dose spillage to the lung and heart [6]. Quantitative analysis of normal tissue effects in the clinic (QUANTEC) for lung clearly emphasizes the need to limit the V5Gy to less than <60%, V20Gy less than 30-35% and the mean lung dose (MLD) to < 23 Gy [7]. Improved survival in early breast cancer patients has led the radiation oncology fraternity to focus on reducing the dose to the heart and lungs. Emerging data has suggested a reduction of radiation-induced cardiac toxicity with modern CT-based planning techniques [8]. Darby, et al. [9] conducted a population-based case-control study to assess the risk of ischemic heart disease in women after radiotherapy for breast cancer. They cautioned about the potential risk for cardiac injury even at low doses, with a relative risk of 7.4% per Gray increase in adverse cardiac events. This is the first planning case reported in the literature on h-VMAT for synchronous bilateral breast cancer patients.

## Conclusions

In the present planning case report, both h-VMAT and VMAT achieved acceptable target coverage while avoiding the field overlapping issues. The h-VMAT achieved better sparing of lungs and heart at low dose region. Since lung fibrosis and cardiac events often manifest 20-30 years post treatment, it is vital that patients have robust long-term clinical follow-up to determine the actual dosimetric benefits of these innovative radiotherapy techniques.
